# Effect of Physical Exercise on Cognitive Function of Alzheimer's Disease Patients: A Systematic Review and Meta-Analysis of Randomized Controlled Trial

**DOI:** 10.3389/fpsyt.2022.927128

**Published:** 2022-06-16

**Authors:** Wei Liu, Jia Zhang, Yanyan Wang, Junfeng Li, Jindong Chang, Qingyin Jia

**Affiliations:** ^1^School of Physical Education, Xuzhou Kindergarten Teachers College, Xuzhou, China; ^2^Institute of Motor Quotient, Southwest University, Chongqing, China; ^3^Qingdao Mental Health Center, Qingdao University, Qingdao, China; ^4^Ministry of Sports, Shandong Technology and Business University, Yantai, China; ^5^Financial Department, Shandong Sports University, Jinan, China

**Keywords:** Alzheimer's disease, cognition, physical exercise, systematic review, meta-analysis

## Abstract

This review aims to systematically review the effects of physical exercise on the cognitive performance of patients with Alzheimer's disease (AD) and its mechanisms of action. Databases such as Web of Science, PubMed, EMBASE, and the Cochrane Central Register of Controlled Trials were searched until December 2021. A randomized controlled trial (RCT) to assess the effect of an exercise intervention (compared with no exercise) on patients with AD. The measures included cognitive function [Mini-Mental State Examination (MMSE), Alzheimer's Disease assessment scale-cognitive (ADAS-Cog), Montreal cognitive assessment scale (MoCA) and Executive Function (EF)]. The methodological quality of the included literature was assessed using the Physiotherapy Evidence Database (PEDro) scale. Twenty-two studies (*n* = 1647, mean age: 77.1 ± 6.3 years) were included in the systematic review, sixteen of which were included in the meta-analysis. A systematic review and meta-analysis revealed that physical exercise positively affects cognitive performance in older patients with AD. However, the positive effects of the intervention should be interpreted with caution considering the differences in methodological quality, type, frequency, and duration of exercise in the included studies. Future studies should consider the design rigor and specification of RCT protocols.

## Introduction

Alzheimer's disease (AD) is a chronic neurodegenerative disease that has no known treatable cure (1). As the disease gradually destroys brain structures (e.g., hippocampus and internal olfactory cortex) (2), it leads to loss of cognitive mental functions, including memory, language, attention, and perception, reduced activities of daily living, and diminished quality of life (3).

The global increase in the prevalence of AD is closely linked to the aging of the population (4). According to the World Alzheimer's Disease Report 2021, more than 55 million people worldwide are living with cognitive impairment, which is expected to reach 78 million by 2030 (5). The direct cost to American society of caring for patients with AD was estimated at $305 billion in 2020 and was expected to exceed $1 trillion by 2050 (6). The high cost of treatment prevents 75% of people with dementia worldwide from being effectively diagnosed and treated (5).

Although not all studies support it, there is growing evidence that physical exercise can prevent cognitive decline and dementia (7–10). Physical exercise, such as aerobic (11–13), stretching (14), resistance (15), or combined exercises (16–20), may delay and prevent cognitive decline in older adults with AD. Therefore, physical exercise emerges as one of the most promising, effective, and least expensive strategies to prevent and delay cognitive decline in patients with AD (21, 22).

Numerous studies have shown that physical exercise is associated with positive effects on brain health (23–25). High levels of aerobic exercise have been associated with improved brain volume and factors of cognitive decline (26, 27). Some studies have shown that aerobic exercise can boost brain plasticity (28), reduce hippocampal atrophy (29), and even increase the hippocampus (30). Physical exercise appears to affect brain atrophy positively in older adults with AD (31). Cognitive impairment is one of the forms of brain atrophy presentation, which results in difficulty in controlling physical mobility in patients with AD (32). Regular exercise at an appropriate intensity and physically demanding level may stimulate some cognitive functions in older adults with AD (33). Therefore, physical exercise appears to be one of the active strategies to resist brain atrophy in older adults with AD.

Several reviews have been published on the effects of physical exercise on the cognitive performance of older adults with AD (19, 34–37). Some reviews concluded that the intervention was beneficial for global cognitive impairment (19, 35). Others concluded that the intervention effect was not beneficial (36); some meta-analyses concluded that physical exercise improved cognition in the AD group with an effect comparable to donepezil (38). By way of example, physical exercise can improve cognition. Previous studies have confirmed that physical activity can improve cognition in AD patients, but the dose-effect relationship between physical activity and AD is not clear. Moreover, systematic reviews are lacking for analyzing the type, intensity, frequency, and duration of physical exercise interventions in patients with AD.

**Figure 1:**
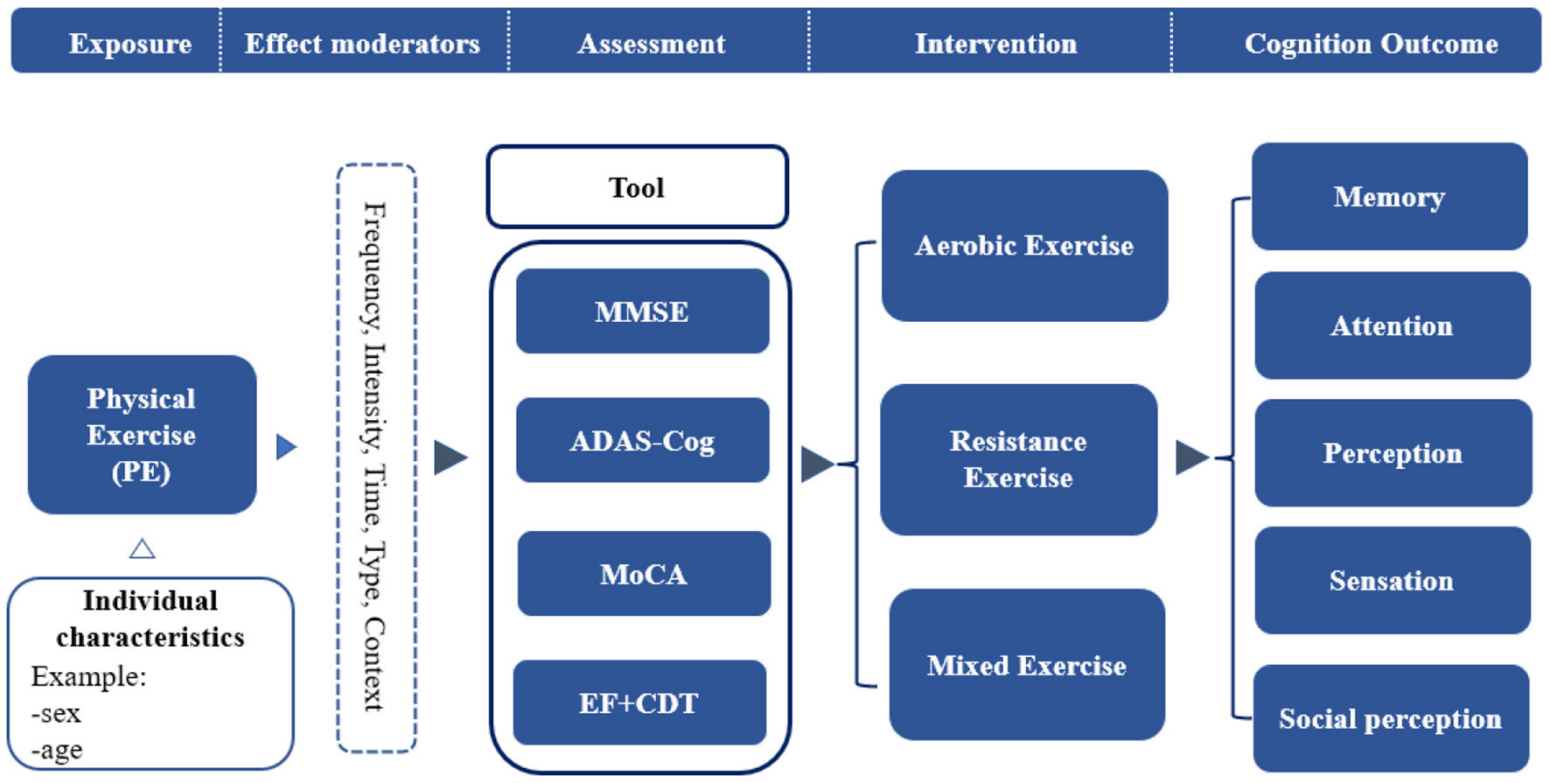
A conceptual model of physical exercise intervention for cognitive performance.

Therefore, the purposes of this review were (1) to systematically review the effects of physical exercise on the cognitive performance of patients with AD; (2) to determine the dose-effect of physical exercise on patients with AD, and (3) to explore the mechanisms underlying the effects of physical exercise on the cognitive performance of patients with AD. To this end, we developed a conceptual model of physical exercise interventions for the cognitive performance of AD patients (Figure 1).

## Methods

### Data Sources and Search Strategies

The study of this systematic review and meta-analysis followed the PRISMA guidelines (39). Three databases, including Web of Science, PubMed, EMBASE, and the Cochrane Central Register of Controlled Trials, were searched for literature written in English only. The search terms used include “Alzheimer's” or “Alzheimer's disease” or “AD” or “dementia” and “exercise” or “physical exercise” or “aerobic exercise” or “resistance training” and “cognitive function” or “executive function.” The search strategy was determined by three investigators, with two investigators working independently on the search task and the third involved in resolving any search disputes. The search covered the period from creating the database to December 31, 2021.

### Study Selection

The criteria for inclusion in the study were (1) design: randomized controlled trial (RCT); (2) sample: at least one group of participants with a diagnosis of Alzheimer's disease type dementia in older adults (mean age 65 years or older), excluding other diagnoses of dementia or MCI; (3) intervention: aerobic, resistance, or stretching type of physical exercise was performed; (4) outcome: at least one executive function or cognitive function was measured. The following studies were excluded from systematic evaluation: (1) non-interventional studies; (2) non-exercise type studies of intervention modalities; (3) theoretical studies, descriptions of treatments, or methodological protocols; (4) review articles; and (5) non-English language articles.

### Data Extraction

Two investigators (WL and JL) retrieved and collected data, and potential disagreements were resolved by joint discussion with two other investigators (JC and QL). Data collected from each study included subject characteristics (mean age, number of genders), intervention protocol (exercise type, frequency, duration, intensity), indicators of cognitive outcomes, intervention effects, and pre- and post-intervention outcomes (expressed as mean ± standard deviation). The conversion was performed using the formula $SD=SEM∙\sqrt{n}$ for studies that provided standard error of the mean (SEM) for outcomes. When the outcomes data were expressed as mean and confidence interval (CI), the formula for conversion of CI to SD was $SD=\frac{upper\text{ limit}-lower\text{ limit}}{3.92}∙\sqrt{n}$ (40). If some studies only displayed graphs containing means and standard deviations, the GetData Graph Digitizer was used for digitizing and extracting the data (41). All studies included in the meta-analysis were used for data synthesis, regardless of their methodological quality.

### Qualitative Analysis

The methodological quality assessment of each study was conducted independently by two reviewers (JZ and YW) using the Physiotherapy Evidence Database (PEDro) Scale (42). The PEDro scale was developed to assess the quality of a treatment or intervention study design, including assessment of randomization, blinding, attrition, design, and statistics. According to the PEDro scale scoring rules, each item was scored independently, with “yes” being scored as “1” and “no” or “unclear” as “0,” and the maximum score for the ten criteria was 10. The possible risk of bias was determined from the extracted information, with <5/10 being rated as “high” risk and more than 5/10 as “low” risk (43). If the details in the article were unclear, we judged the risk of bias as “unclear” and contacted the corresponding author for more information. If the corresponding author did not provide clarification within ten working days, the item was scored as “0.”

### Statistical Analysis

Review Manager Software V.5.3 was used for statistical analysis of the combined data. Statistical significance was defined for bilateral *p* <0.05. The combined data effects were presented using the mean difference (MD) and the corresponding 95%CI of the continuous effects. If data were available and no significant heterogeneity was detected, a fixed-effects model was used to calculate the combined effect. Otherwise, a random-effects model was applied. Statistical heterogeneity was assessed using the *I*^2^ statistic. However, when heterogeneity between studies was high (*I*^2^ > 75%), overall pooled analysis was considered inappropriate; clinical or methodological heterogeneity was considered a potential cause. Heterogeneity between studies was explored using the χ2 test and Higgins *I*^2^ values (44). Studies with different intervention types were divided into subgroups for analysis based on different factors, given the potential heterogeneity between studies.

## Results

### Study Selection

**Figure 2:**
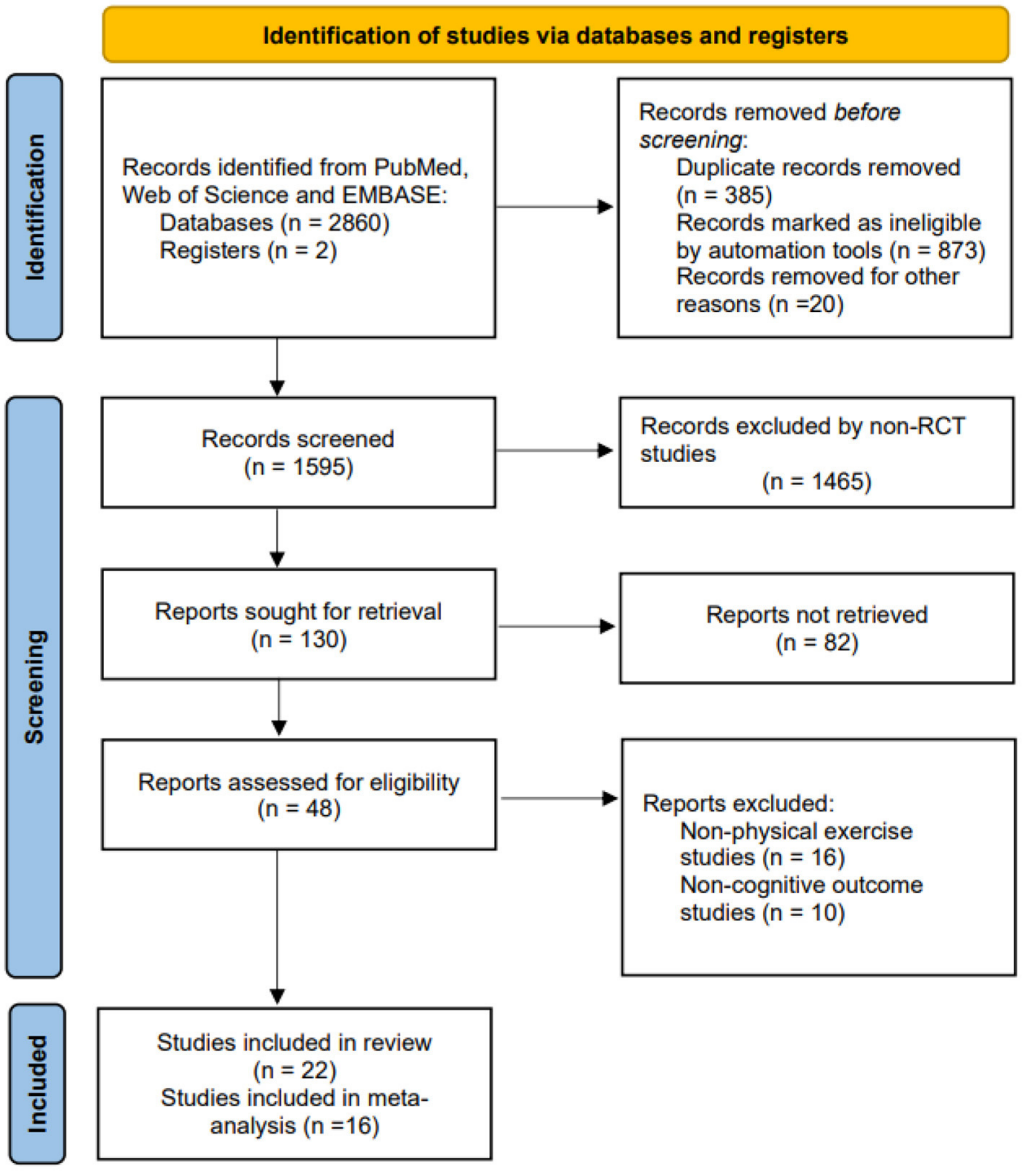
PRISMA flowchart of included and excluded studies.

A total of 2,862 literature records were initially identified according to the proposed search strategy. Two investigators (WL, JL) screened by abstract and title, and apparently irrelevant records were excluded. A total of forty-eight potential studies were included for further evaluation. Of these, sixteen studies that did not provide physical exercise and ten studies that did not involve cognitive outcomes were excluded. Finally, twenty-two studies were eligible for inclusion in the systematic review, and the data from sixteen studies were extracted for meta-analysis. The detailed literature selection and screening process are described in Figure 2.

### Study Characteristics

The characteristics of selected studies are detailed in Table 1. A total of twenty-two RCTs involving 1,647 AD participants (age 77.1 ± 6.3 years) were included in the study for review. The included studies were from sixteen countries, namely Brazil (16, 45, 47), France (18, 46, 48), USA (50, 54, 56), Italy (13, 53), Nigeria (17), Netherlands (49), Denmark (11), Germany (15), Egypt (12), Finland (19), Iran (51), Albania & Spain (14), Sweden (52), Australia (20), and China (55). Participants were from the community, social centers, social clubs or individuals, memory clinics, or hospitals. All studies reported identified inclusion and exclusion criteria for the diagnosis of AD participants. The study interventions contained aerobic, resistance, stretching, or mixed exercise (aerobic, resistance, balance, and stretching exercises). Nine studies performed a single aerobic training session, including supervised aerobic, walking, or cycling training (11–13, 48, 50, 53–56). Eleven studies used mixed exercise modalities, combining aerobic, resistance, balance, stretching, or cognitive training (16–20, 45–47, 49, 51, 52). Besides, two studies were conducted on resistance exercise (15) and stretching exercise (14), respectively.

**Table 1:** Characteristics of the inclusion studies.

**ID**	**Author, year (Country)**	**Age (years)**	**Subjects male/all**	Duration (min*tim*wk)	**Intensity**	**Intervention exercise**	**Exercise categories**	**Cognitive outcome**	**Effect**
1	Aguiar (45) (Brazil)	EG: 78.6 ± 8.4 CG: 74.7 ± 7.4	9/34	40*2*20	NA	Interchangeably between sessions A (aerobic activity) and B (resistance exercise and balance training)	ME (AE, RE, BE)	MMSE	Cognition remained unchanged in both groups
2	Arcoverde (16) (Brazil)	EG: 78.5 CG: 79	NA/20	30*2*16	60% VO_2_max	Treadmill, stretching, big muscles groups	ME (AE, RE, FE)	MMSE CDT (EF)	EG showed improvement in cognition
3	Barreto (46) (France)	EG: 88.3 ± 5.1 CG: 86.92 ± 5.8	14/91	60*2*24	Moderate intensity	Multicomponent training: coordination and balance exercises, muscle strengthening, aerobic exercise (mostly walking)	ME (AE, RE, BE, CE)	MMSE	Cognitive function did not display differences
4	De Oliveira Silva (47) (Brazil)	EG: 81.22 ± 8.88 CG: 77.54 ± 8.05	11/27	60*2*12	70% VO_2_max or 80% MHR	Multimodal training: balance, aerobic, and strength training and stretching	ME (AE, RE, BE, FE)	MMSE CDT (EF)	Cognition improvements were not observed in patients with AD
5	Enette (48) (France)	77.9 ± 7.6	19/52	30*2*9	EG1: 70% MHR, moderate EG2: 80% MHR, vigorous	EG1: continue aerobic training EG2: interval aerobic training	AE	MMSE	No significant change in cognitive performance after interventions
6	Gbiri (17) (Nigeria)	EG: 68.7 ± 3.4 CG: 70.6 ± 3.3	14/31	70*2*12	Each station 80% start, 10% progression (interval)	Motor functions, gait, posture, cognition, balance and productivity	ME (FE, BE)	MMSE ADAS-Cog	A significant improvement in cognition
7	Henskens (49) (The Netherlands)	EG1: 87.0 ± 7.2 EG2: 86.1 ± 5.9 EG3: 85.1 ± 4.6 CG: 84.7 ± 4.6	20/87	30-45*3*26	Progressive increase	Alternating strength and aerobic sessions	ME (AE, RE)	MMSE EF	No significant effects in cognitive functions
8	Hoffmann (11) (Denmark)	EG: 69.8 ± 7.4 CG: 71.3 ± 7.3	113/200	60*3*16	Moderate-to-high intensity, 70–80% MHR	Aerobic exercise in the ergometer bicycle, cross trainer, and treadmill	AE	MMSE ADAS-Cog	Neuropsychiatric symptoms were significantly less severe
9	Holthoff (15) (Germany)	EG: 72.4 ± 4.3 CG: 70.7 ± 5.4	15/30	30*3*12	NA	Trained lower body on a movement trainer	RE	MMSE EF	Executive function improvement in the EG
10	Kemoun (18) (France)	81.8 ± 5.3	NA/31	60*3*15	Light to moderate, 60–70% FCR	Walking, equilibrium and stamina exercises	ME (AE, BE)	ERFC	Intervention group improved, while the control group decreased
11	Morris 2017(50) (USA)	EG: 74.4 ± 6.7 CG: 71.4 ± 8.4	37/78	30-50*3-5*26	From 40–55% to 60–75% of HRR	Aerobic exercise course	AE	EF	No clear effect of intervention on Memory and Executive Function
12	Nagy (12) (Egypt)	65-73	30/60	45-60*3*12	Moderate-intensity 40–50% HRR ~ 50–70% HRR	Aerobic exercise	AE	MoCA	A statistically significant differences were observed in MoCA–B scores
13	Ohman (19) (Finland)	EG1: 77.7 ± 5.4 EG2: 78.3 ± 5.1 CG: 78.1 ± 5.3	129/210	60*2*52	Low intensity	Aerobic, strength & endurance, balance, and executive functioning training	ME (AE, RE, BE)	MMSE CDT (EF)	Executive function was improved in the HE group
14	Parvin (51) (Iran)	67.4 ± 8.8	NA/26	40-60*2* 12	NA	Progressive combined exercises with visual stimulation, including muscle endurance, balance, flexibility, and aerobic exercises with eyes closed and opened	ME (AE, BE, FE)	MoCA	A significant improvement in cognitive function, particularly in short-term and working memory, attention, and executive function
15	Todri (14) (Albania & Spain)	81.7 ± 5.24	40/90	40*3*24	NA	Stretching and respiratory exercise	FE	MMSE	A significant effect on the difference between groups
16	Toots (52) (Sweden)	85.1 ± 7.1	45/186	45*2.5*16	SE: 40%MI & 45% HI BE: 27%MI & 63% HI	High Intensity Functional Exercise (HIFE) program	ME (AE, RE, FE)	MMSE ADAS-Cog	No superior effects on global cognition or executive function
17	Venturelli (13) (Italy)	EG: 83 ± 6 CG: 85 ± 5	NA/21	30*4*24	NA	Supervised walking aerobic exercise	AE	MMSE	EG did not show a significant improvement in cognition, CG showed a significant decreased
18	Venturelli (53) (Italy)	EG1: 84 ± 7 CG: 84 ± 10	22/80	60*5*12	Moderate intensity	Walking	AE	MMSE	No significant changes in cognitive function (MMSE scores)
19	Vidoni (54) (USA)	EG: 71.2 ± 4.8 CG: 72.2 ± 5.3	38/117	30-50*3-5* 52	Moderate-intensity 40–50% HRR ~ 50–70% HRR	Supervised aerobic exercise	AE	EF	No effect of aerobic exercise on cognitive measures
20	Vreugdenhil (20) (Australia)	74.1(51-89)	16/40	30-60*7*16	NA	Walking, upper and lower body strength and balance training	ME (AE, RE, BE)	MMSE	Cognitive function improved in EG
21	Yang (55) (China)	EG: 72.0 ± 6.7 CG: 71.9 ± 7.3	33/50	40*3*12	70% MHR	Cycling training	AE	MMSE ADAS-Cog	Cognitive function improved in aerobic group
22	Yu (56) (USA)	77.4 ± 6.8	53/86	40-60*3*24	EG: 50–75% HRR CG: <20% HRR	EG: cycling exercise; CG: stretching	AE	ADAD-Cog EF	A significantly less than the natural increase in cognitive function

*min, minute; tim, time; wk, week; NA, Not Applicable; VO_2_max, maximal oxygen consumption; MTP, Maximal Tolerated Power; MHR, maximum heart rate; HRR, heart rate reserve; MI, moderate intensity; HI, high intensity; FCR, reserve cardiac frequency; ME, mixed exercise; AE, aerobic exercise; RE, resistance exercise; BE, balance exercise; CE, coordination exercise; FE, flexibility exercise; ADAS-Cog, Alzheimer's Disease assessment scale-cognitive; MMSE, mini-mental state examination; MoCA, Montreal cognitive assessment scale; CDT, Clock draw test; EF, Executive Function; ERFC, Rapid Evaluation of Cognitive Functions test (French version)*.

The frequency of interventions ranged from 2 to 5 times per week for 30–70 min each. The duration of the interventions ranged from 9 to 52 weeks. Participants had heart rate reserve (HRR) from 40 to 80%, maximum oxygen uptake (VO2max) from 60 to 70%, and maximum heart rate (MHR) from 60 to 80% in the inclusion studies. Furthermore, six studies did not describe the intensity of intervention exercise (13–15, 20, 45, 47). The control group interventions generally utilized low-intensity activities/exercises such as social activities, stretching exercises, or health education.

Meta-analysis was used to analyze cognitive outcomes, including cognitive functions, mental health, and executive functions. Various measurement tools were used to assess studies in the same or similar cognitive domains, including the MMSE, ADAS-Cog, MoCA, CAMCOG, CDT, and others. Of the studies included in the meta-analysis, thirteen studies measured the MMSE (11, 13–20, 45–47, 49, 52, 53, 55), four measured the ADAS-Cog (20, 52, 55, 56), six measured the executive function (EF+CDT) (16, 19, 49, 50, 54, 56), and two measured the MoCA (12, 51).

### Quality Assessment

Twenty-two studies included in the systematic review were identified with the PEDro score to assess methodological quality. Only one study had a PEDro score of 5, and the other twenty-one studies had a PEDro score of ≥6, indicating good quality. Nevertheless, we also found fewer studies on participant and therapist blinding in the PEDro quality assessment. Only three studies blinded participants, and only one study blinded therapists. Details of the raw records are shown in Table 2.

**Table 2:** Assessment of quality of study design using PEDro.

**ID**	**Study**	**Eligibility**	**Random**	**Concealed**	**Groups similar**	**Participants**	**Therapist**	**Assessor**	** <15%**	**Intension**	**Between**	**Point estimate**	**Total**
		**and source**	**allocation**	**allocation**	**at baseline**	**blinding**	**blinding**	**blinding**	**dropouts**	**-to- treat**	**-group**	**and variability**	
										**analysis**	**difference**	**reported**	
											**reported**	**reported**	
1	Aguiar 2014	Y	Y	Y	Y	N	N	Y	Y	Y	Y	Y	8/10
2	Arcoverde 2014	Y	Y	Y	Y	N	N	N	Y	Y	Y	Y	7/10
3	Barreto 2017	Y	Y	Y	Y	Y	N	Y	Y	Y	Y	Y	9/10
4	De Oliveira Silva 2019	Y	Y	Y	Y	Y	N	Y	Y	Y	Y	Y	9/10
5	Enette 2020	Y	Y	N	Y	Y	N	N	Y	Y	Y	Y	7/10
6	Gbiri 2020	Y	Y	N	Y	N	Y	Y	Y	Y	Y	Y	8/10
7	Henskens 2018	Y	Y	Y	Y	N	N	N	Y	Y	Y	Y	7/10
8	Hoffmann 2016	Y	Y	Y	Y	N	N	Y	Y	Y	Y	Y	8/10
9	Holthoff 2015	Y	Y	N	Y	N	N	N	Y	Y	Y	Y	6/10
10	Kemoun 2010	Y	Y	N	Y	N	N	N	Y	Y	Y	Y	6/10
11	Morris 2017	Y	Y	Y	Y	N	N	Y	Y	Y	Y	Y	8/10
12	Nagy 2021	Y	Y	Y	Y	N	N	N	Y	Y	Y	Y	7/10
13	Ohman 2016	Y	Y	Y	Y	N	N	N	Y	Y	Y	Y	7/10
14	Parvin 2020	Y	Y	Y	Y	N	N	N	Y	Y	Y	Y	7/10
15	Todri 2019	Y	Y	Y	N	N	N	Y	Y	Y	Y	Y	7/10
16	Toots 2017	Y	Y	Y	Y	N	N	N	Y	Y	Y	Y	7/10
17	Venturelli 2011	Y	Y	N	Y	N	N	Y	Y	Y	Y	Y	7/10
18	Venturelli 2016	Y	Y	N	Y	N	N	Y	Y	Y	Y	Y	7/10
19	Vidoni 2021	Y	Y	Y	Y	N	N	Y	Y	Y	Y	Y	8/10
20	Vreugdenhil 2012	Y	Y	N	Y	N	N	Y	Y	Y	Y	Y	7/10
21	Yang 2015	Y	Y	N	Y	N	N	N	Y	Y	N	Y	5/10
22	Yu 2021	Y	Y	Y	Y	N	N	N	Y	Y	Y	Y	7/10

### Effects of Interventions

A total of thirteen of the included studies showed significant cognitive and executive functions changes (11–17, 19, 20, 51, 53, 55, 56), and nine studies showed no significant differences in cognitive and executive functions (45–50, 52–54).

**Figure 3:**
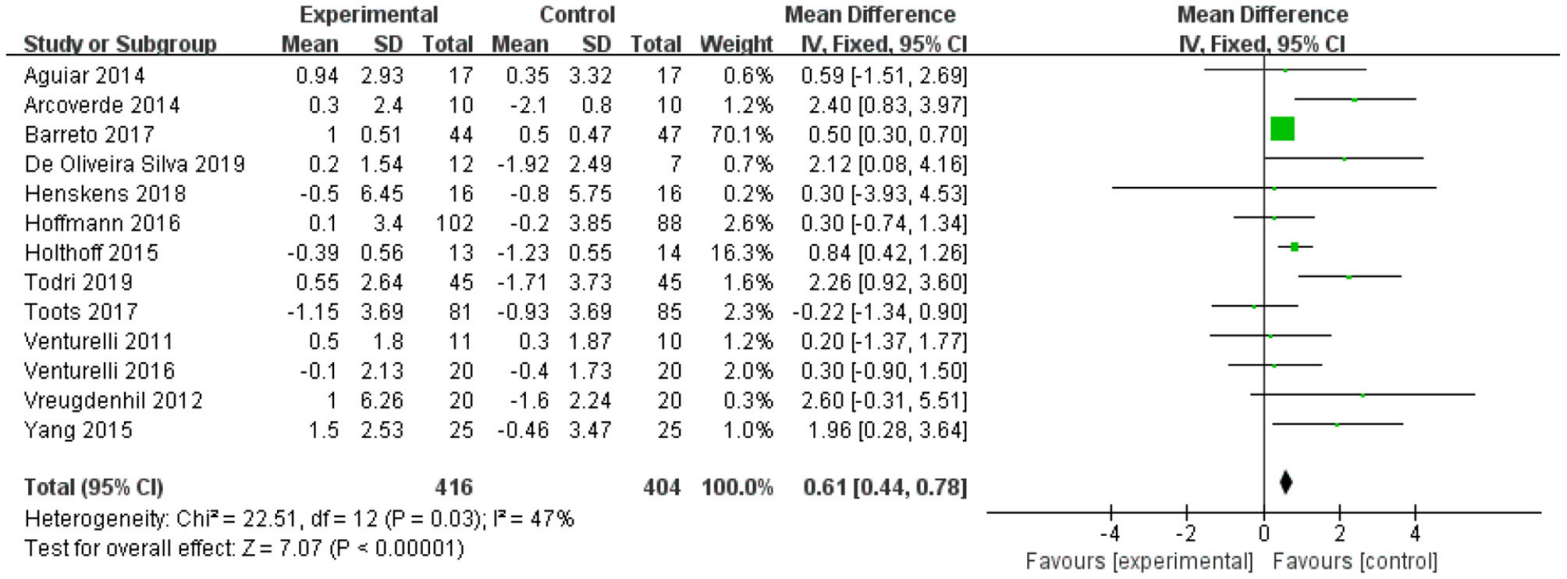
Forest plot for physical exercise interventions on cognition function (MMSE).

Thirteen studies involving 820 participants measured the effect of physical exercise on cognitive function in patients with AD using the MMSE (11–20, 45–47, 49, 52, 53, 55). The meta-analyses showed a significant improvement in cognitive function with physical exercise (*n* = 820, MD = 0.83, 95% CI = 0.43 to 1.23, *p* <0.00001; *I*^2^ = 47%, fixed-effect model; Figure 3).

**Figure 4:**
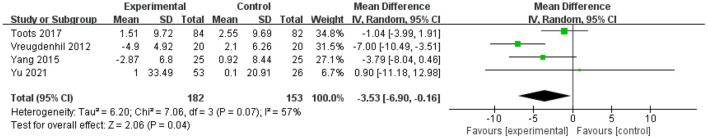
Forest plot for physical exercise interventions on cognition function (ADAS-Cog).

Four studies assessed the effect of physical exercise on cognitive function using the ADAS-Cog (20, 52, 55, 56). The meta-analysis showed a significant improvement in MD scores of cognitive functions with physical exercise (*n* = 335, MD = −3.533, 95% CI = −6.9 to −0.16, *p* = 0.04; *I*^2^ = 57%, random-effect model; Figure 4).

**Figure 5:**
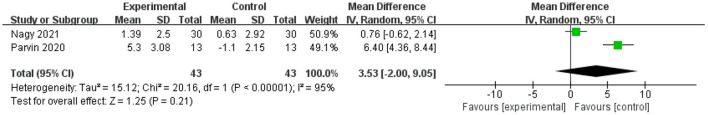
Forest plot for physical exercise interventions on cognition function (MoCA).

Two studies were performed to assess the effect of physical activity on cognitive function using MoCA (12, 51). Both studies showed a positive effect of physical exercise on cognitive function. Nevertheless, the meta-analysis showed no significant improvement in MD scores of cognitive functions (*n* = 86, MD = 3.53, 95% CI = −2.00 to 9.05, *p* = 0.21; *I*^2^ = 95%, random-effects model; Figure 5).

**Figure 6:**
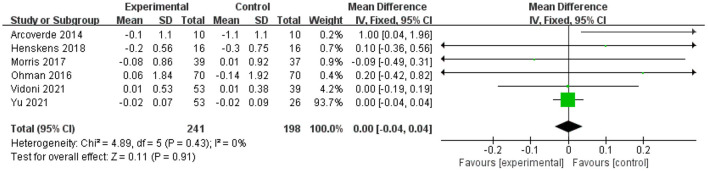
Forest plot for physical exercise interventions on execution function.

Six studies analyzed the effects of physical exercise on executive function (16, 19, 49, 50, 54, 56). The meta-analysis showed no significant improvement in MD scores of cognitive functions with physical exercise (*n* = 439, MD = 0, 95% CI = −0.04 to 0.04, *p* = 0.91; *I*^2^ = 0%, fixed-effects model; Figure 6).

## Discussion

This review examined the effects of physical activity on the cognitive function of older patients with AD. Although 41% of the studies in the systematic review did not show a positive intervention effect, there was a significant improvement in the cognitive performance of patients in 59% of the studies. In the meta-analysis, the studies using the MMSE (*p* <0.0001) and ADAS-Cog (*p* = 0.04) measures of cognitive performance both showed significant improvements, while the studies using the MoCA (*p* = 0.21) and EF (*p* = 0.91) tests did not show significant improvements. Thus, we identified that physical exercise interventions are beneficial for improving cognitive function in AD patients.

Several previous meta-analyses reported a significant positive effect of physical exercise interventions on attenuating a cognitive decline in patients with AD (57–59). The present study was consistent with using the MMSE test for significant positive effects of exercise on cognitive performance interventions. In this study, the percentage of significant studies was higher when analyzing aerobic exercise separately than mixed exercise. For example, aerobic exercise interventions were significant in 67% of the included studies; mixed exercise was 55%. Notably, the benefits of aerobic exercise on cognitive performance in patients with AD appear to be at least similar to the minimal clinically significant differences (MCID) reported in previous studies (60).

Although the findings of this study meta-analysis support the effect of physical exercise on cognitive improvement in patients with AD, it is difficult to determine whether the cognitive improvement is due to the type of exercise (aerobic, resistance, stretching, or mixed), the amount of exercise, or the intensity of exercise. This review includes three exercise types: aerobic exercise, resistance exercise, stretching exercise, and mixed exercise. Both resistance and stretching exercises separately showed a positive effect of the intervention. However, 45% of the studies in mixed exercise did not show a positive effect of the intervention. Moreover, the least amount of exercise in all studies was 540 min (48), and the most were 7,800 min (54), and neither study found a significant cognitive improvement effect. Therefore, this means that the relationship between the amount of exercise and the effect of exercise is not clear. As for exercise intensity, of the fifteen studies in the included literature that involved moderate exercise intensity, 47% had positive intervention effects, while 53% had insignificant effects (Table 1). It remains to be verified that moderate exercise intensity is the recommended criterion, as mentioned in previous studies (61, 62).

The strength of this systematic review is the methodological design, in which the construction of a conceptual model is methodologically focused on the study of the mechanisms of physical exercise (63). First, we propose a “conceptual model of physical exercise interventions for the cognitive performance of patients with AD.” Second, the present study focused on different types of physical exercise, including the intensity, duration, and categories of exercise. In addition, the meta-analysis included randomized controlled trials to ensure the quality of the study literature. The current study also has some limitations. First, the included studies used different measurement instruments and had methodological compatibility issues, which may affect our interpretation of data integration and findings. Second, heterogeneity exists across intervention characteristics, including type, intensity, frequency, and duration of exercise. The type of included studies varied, such as aerobic exercise, resistance exercise, stretching exercise, or mixed exercise; the duration of each exercise session ranged from 30 to 70 min, and the duration ranged from 9 to 52 weeks. Therefore, the optimal design of intervention studies remains unclear, and further research is necessary. Third, the different levels of quality of the included studies and the methodological heterogeneity may lead to our interpretation of the results. Fourth, based on the current systematic review and meta-analysis of studies, we found few studies reporting analysis of the effects of combined interventions (only two), such as physical exercise with cognitive training interventions and physical exercise with pharmacological interventions. We will focus on the above issues in future studies. Additionally, since it is not possible to blind participants in physical exercise intervention experiments; therefore, such bias in the study design may exist.

## Conclusion

Physical exercise interventions effectively improve cognitive performance in older patients with AD, which may indicate the potential value of physical exercise in improving cognitive performance and preventing conversion to severe dementia in patients with AD. However, considering the differences in methodological quality, type, frequency, and duration of exercise in the included studies, the positive effects of the intervention should be interpreted with caution. More rigorous designs and standardized RCT protocols will be considered for future studies.

## Data Availability Statement

The original contributions presented in the study are included in the article/supplementary materials, further inquiries can be directed to the corresponding author/s.

## Author Contributions

WL and JL: data collection. JZ and YW: data analysis, conception, and design. WL, JL, JC, and QJ: research design, writing the manuscript, and revision. All authors contributed to the article and approved the submitted version.

## Funding

This study was funded by the Humanities and Social Sciences Project of the Ministry of Education of China (No. 17YJC890020), the Ability Promotion Program of the Fundamental Research Funds for the Central Universities of Southwest University (No. SWU2209230), the Humanities and Social Sciences Planning Project of Chongqing Municipal Education Commission (No. 20SKGH046), and the Teaching Reform Project of Southwest University (No. 2021JY071).

## Conflict of Interest

The authors declare that the research was conducted in the absence of any commercial or financial relationships that could be construed as a potential conflict of interest.

## Publisher's Note

All claims expressed in this article are solely those of the authors and do not necessarily represent those of their affiliated organizations, or those of the publisher, the editors and the reviewers. Any product that may be evaluated in this article, or claim that may be made by its manufacturer, is not guaranteed or endorsed by the publisher.
